# Advances in platinum-based and platinum-free oxygen reduction reaction catalysts for cathodes in direct methanol fuel cells

**DOI:** 10.3389/fchem.2022.1073566

**Published:** 2022-11-17

**Authors:** Chu Qin, Shijun Tian, Wenjie Wang, Zhong-Jie Jiang, Zhongqing Jiang

**Affiliations:** ^1^ Key Laboratory of Optical Field Manipulation of Zhejiang Province, Department of Physics, Zhejiang Sci-Tech University, Hangzhou, Zhejiang, China; ^2^ Guangdong Engineering and Technology Research Center for Surface Chemistry of Energy Materials and Guangzhou Key Laboratory for Surface Chemistry of Energy Materials, New Energy Research Institute, College of Environment and Energy, South China University of Technology, Guangzhou, Guangdong, China

**Keywords:** direct methanol fuel cells, ORR catalysts, cathodes, platinum-based, carbon supports

## Abstract

Direct methanol fuel cells (DMFCs) have been the focus of future research because of their simple structure, abundant fuel sources, high energy conversion efficiency and low cost. Among the components in DMFC, the activity and stability of the cathode catalyst is the key to the performance and lifetime of the DMFCs. Oxygen reduction reaction (ORR) is an important electrode reaction on DMFC cathode. It is known that Pt is widely used in the fabrication of ORR catalysts, but the limited earth storage of Pt and its high price limit the use of Pt-based commercial catalysts in DMFCs. To overcome these problems, advances have been made on new low Pt-based catalysts and Pt-free catalysts in recent years. In this article, the development of novel ORR catalysts and the carbon supports is reviewed and discussed.

## Introduction

With the emerging environmental problems and increasing energy demand, research on clean and sustainable power generation technologies has become more critical for the upcoming energy crisis. Among the many energy storage and conversion technologies, fuel cell is a promising technology with wide potential applications. A fuel cell is an open thermodynamic system that operates by electrochemical reactions and consumes fuel from external sources. The fuels generally include hydrogen, methanol, ethanol, natural gas, etc. The fuels can provide a large amount of power to the cells with the sufficient chemical energy. Fuel cells can be applied in small-scale or large-scale energy systems, such as mobile power systems, portable electronic devices, transportation and military communication equipment, and combined heat and power (CHP) systems (Mekhilef et al., 2012; Zhang et al., 2012; Wang and Jiang, 2017). In addition, fuel cells can be compatible with renewable energy carriers (e.g., hydrogen) for sustainable energy generation. The flexible modular technology of fuel cells can be scaled from homes to large office buildings and even industrial complexes. The technology of separate power generation and cogeneration can provide energy conversion efficiencies of up to 95%, which can save electricity costs by reducing reliance on centralized power generation, making fuel cells promising alternatives to traditional power generation methods (Dodds et al., 2015; Niakolas et al., 2016).

In typical combustion-based power generation technologies, chemical energy is converted to thermal energy, then to mechanical energy, and lastly to electrical energy. Fuel cells, on the other hand, are electrochemical conversion devices. They combine fuel and oxidant, and convert chemical energy to electrical energy. Compared with the traditional power-generating methods, fuel cells have the following advantages: 1) their energy conversion process is sustainable, green, and efficient; 2) their working process is static, with no noises or vibrations; 3) the power output can be adjusted by controlling the number of single cells to achieve the efficiency that meets practical demand; 4) they have a variety of fuel sources; and 5) they can reduce pollutant emissions. Furthermore, high energy density and portability are significant properties of fuel cells that distinguish them from other power production systems. Fuel cells are better choices in rural areas without access to the public grid or where wiring and transmission costs are too high (Debe, 2012; Sharaf and Orhan, 2014; Stephens et al., 2016).

**FIGURE 1:**
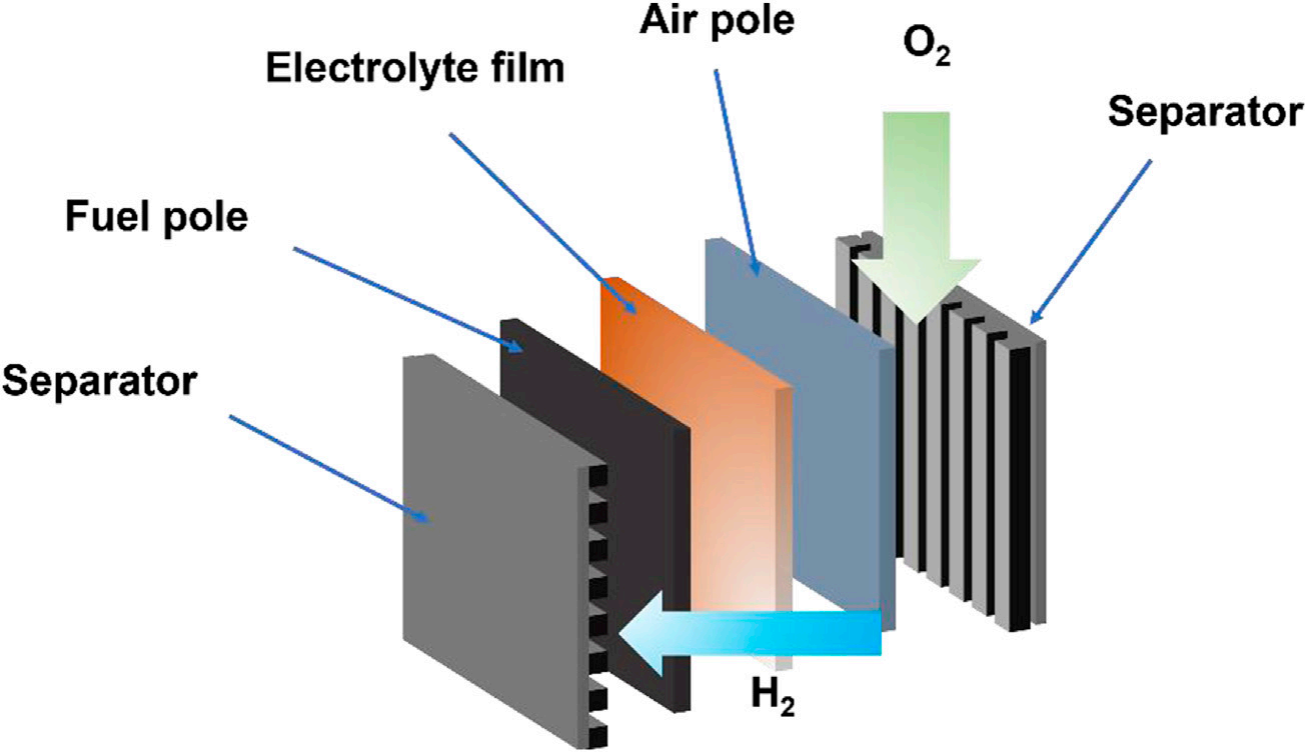
The configuration of a fuel cell.

Although fuel cells, like traditional batteries, convert chemical energy into direct current *via* redox reactions within the battery, they are energy conversion devices rather than energy storage devices. The electrodes of fuel cells are not metals immersed in weak acid but composites composed of proton-conducting medium, carbon-supported catalysts, and electron-conducting fibers. The fuel cells do not need to suffer from electrode material exhaustion; they also do not suffer from cell component failure due to leakage or corrosion (Sharaf and Orhan, 2014). Fuel cells consist of the fuel pole (anode), electrolyte, air pole (cathode), and an external circuit, as shown in Figure 1. In a typical fuel cell, gaseous fuel (e.g., hydrocarbon or hydrogen fuel) and oxidant gas (e.g., oxygen) undergo half-cell reactions at the anode and cathode, respectively. For example, hydrogen undergoes oxidation at the anode to release protons and electrons, and oxygen undergoes a reduction reaction at the cathode, finally producing water. The electrochemical reactions are as shown in Eqs 1–3:

$$2H_{2\left(g\right)}+O_{2\left(g\right)}\to 2H_{2}O+energy$$ (1)

$$Anode：2H_{2}\to 4H^{+}+4e^{-}$$ (2)

$$Cathode：O_{2}+4e^{-}+4H^{+}\to 2H_{2}O$$ (3)

The classification of fuel cells is complicated, as they can be classified by operating temperatures, electrolytes, and fuels. Based on the differences in electrolytes, fuel cells can be commonly divided into polymer electrolyte membrane fuel cells (PEMFC), alkaline fuel cells (AFC), solid oxide fuel cells (SOFC), phosphoric acid fuel cells (PAFC), high temperature molten carbonate fuel cells (MCFC), direct methanol fuel cells (DMFC), and other types (Perry and Fuller, 2002; Kirubakaran et al., 2009; Merle et al., 2011; Hossain et al., 2017; Wang and Jiang, 2017). If classified by temperature, AFC and PEMFC belong to low temperature fuel cells (less than 100°C), PAFC belongs to medium temperature fuel cells, while SOFC and MCFC belong to high temperature fuel cells.

**FIGURE 2:**
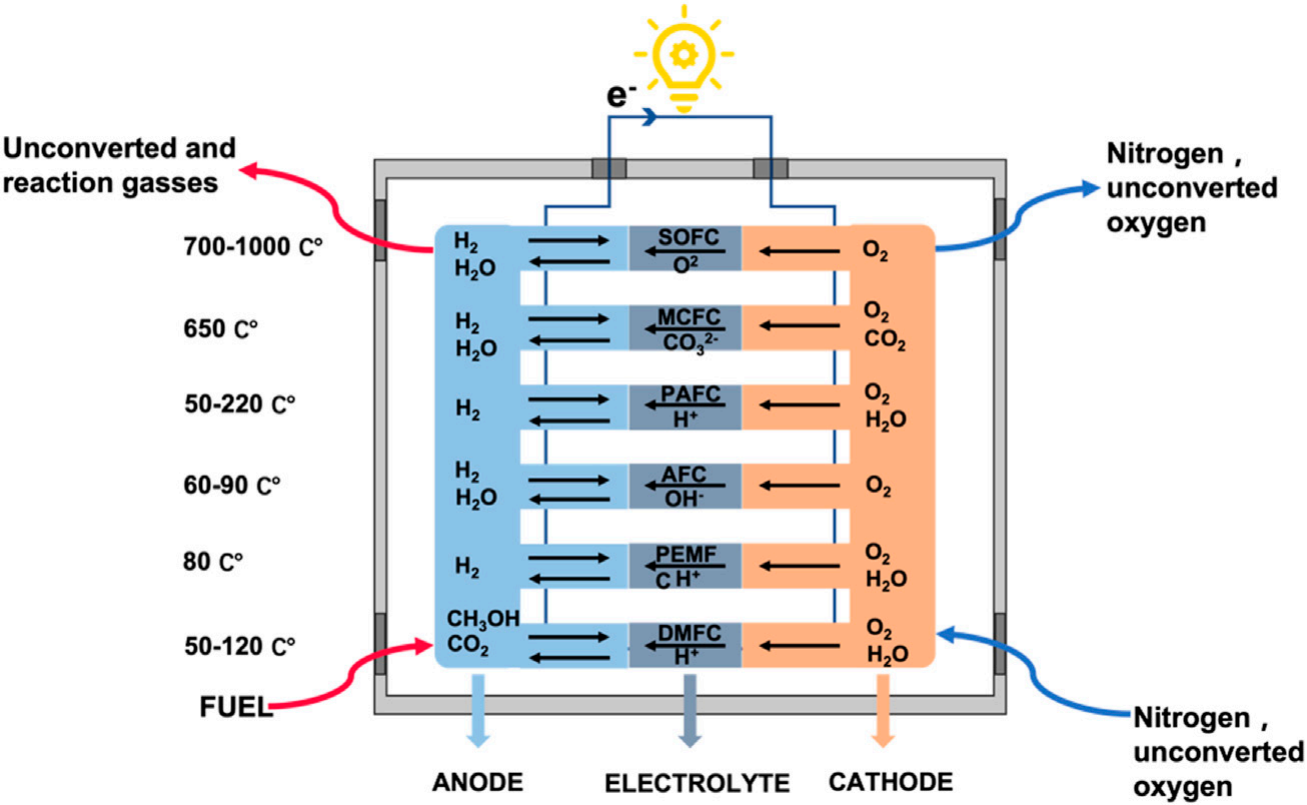
The classification of fuel cells based on the electrolyte, temperature, and fuel types. Adapted under the terms of the Creative Commons CC BY license (Khosravi H et al., 2021). Copyright 2021, Elsevier.

Different types of fuel cells differ in power output, operating temperature, electrical efficiency, and typical applications. In addition, the operating temperature of the fuel cells and the rate of ion transfer also depend on the properties of the electrolytes. Appropriate high temperatures can improve cell efficiency and thus reduce material costs. For example, MCFC and SOFC operating at high temperatures not only generate high heat but also improve fuel efficiency with the extra heat used for hot water supply or internal systems (Haile, 2003; Haydn et al., 2014; Hossain et al., 2017). Figure 2 shows the classification of fuel cells based on the electrolyte, temperatures, and fuel types.

Currently, low temperature fuel cells have been a research hotspot due to their mild operating conditions, like PEMFCs. The problems caused by strong corrosive electrolytes still exist for fuel cells like MCFCs, AFCs, and PAFCs, which hinder their development in the competition with PEMFCs and SOFCs. Using Nafion membranes (a type of conducting polymer) as electrolytes, PEMFCs are the most promising energy conversion technologies in applications such as vehicles, stationary and portable power generation systems due to their low fuel cost, high power density, and energy efficiency (Daud et al., 2004; Chisaka and Daiguji, 2006; Wang et al., 2009b; Nam et al., 2009). Direct methanol fuel cells (DMFCs) are a subcategory of PEMFCs in which methanol rather than hydrogen is consumed in the fuel cells. DMFCs convert chemical energy from liquid fuels into electricity and have been extensively studied in recent years due to their great potential in portable devices, military applications, and vehicles (Ren et al., 2000; Acres, 2001; Aricò et al., 2001; Kamarudin et al., 2009). Compared with other PEMFCs using hydrogen fuel, methanol fuel is favored by the easy storage and transportation conditions as well as the high energy density of methanol in the liquid form (Ren et al., 1996; Scott et al., 1999; Zhao et al., 2009).

**FIGURE 3:**
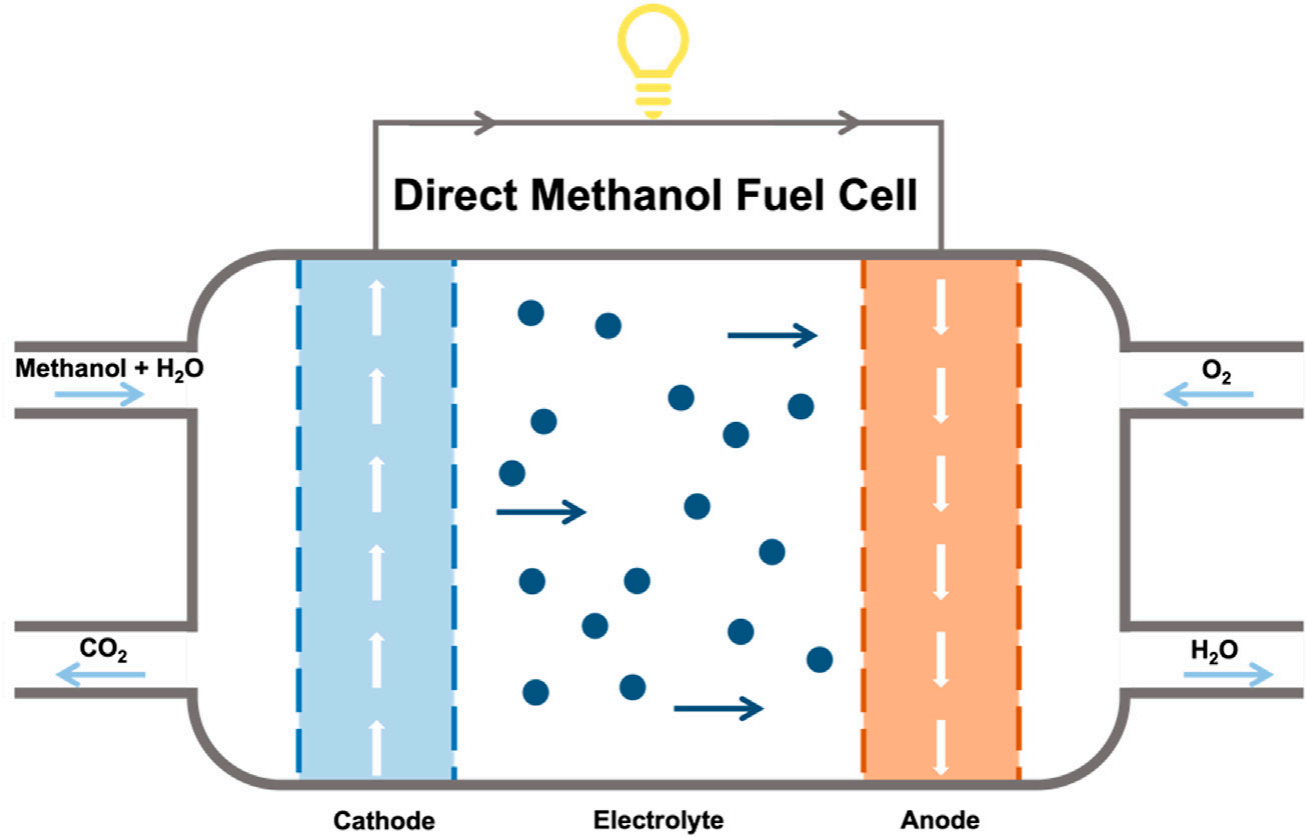
The configuration and components of the DMFC.

The working diagram of DMFC is shown in Figure 3. DMFC consists of the anode, the proton exchange membrane, and the cathode. The anode mainly contains methanol oxidation reaction (MOR) catalysts, and the proton exchange membrane mainly transfers protons (H^+^), and also prevents electrons (e^−^) and methanol (CH_3_OH) from being transferred to the cathode (Liu et al., 2019a; Liu et al., 2019b; Tian et al., 2021; Tian et al., 2022). When the cell is working in an acidic electrolyte, the methanol is transported to the anode and oxidizes to generate CO_2_, protons and electrons, while ORR happens on the cathode at the same time, as shown in Eqs 4–6. The e^−^ is transferred to the anode through the external circuit to generate electricity, while the H^+^ is transferred to the cathode through the electrolyte and proton exchange membrane (Scott et al., 1999; Aricò et al., 2001; Xia et al., 2019).

$$Anode:{CH}_{3}OH+H_{2}O\to {CO}_{2}+6H^{+}+6e^{-}$$ (4)

$$Cathode:\frac{3}{2}O_{2}+6H^{+}+6e^{-}\to 3H_{2}O$$ (5)

$$Overall:{CH}_{3}OH+\frac{3}{2}O_{2}\to {CO}_{2}+2H_{2}O$$ (6)

DMFC is considered the most likely fuel cells to be commercialized due to its advantages of low operating temperature and high energy density. But the commercialization of DMFCs is still restricted by two technical problems. The first is the insufficient activity of cathode catalysts. The Pt/C catalysts commonly used as the cathode catalysts have an overpotential loss of around 200 mV at the open-circuit voltage. The second is methanol crossover through the Nafion membrane. In this case, ORR and MOR will happen on the cathode at the same time, which greatly lowers the efficiency of the DMFC. Even though the polymer membrane is getting improved, high-efficiency and high-selectivity cathode catalysts for ORR are still necessary for practical applications.

For a long time, the slow ORR kinetics has restrained the efficiency and applications of fuel cells (Feng et al., 2019). ORR includes two different reaction pathways based on the property of the medium (Dey et al., 2017; Tian et al., 2020). In an acidic medium, oxygen can be reduced in the four-electron pathway (Eq. 7) or the two-electron pathway (Eqs 8–10):

$$O_{2}+4H^{+}+4e^{-}\to 2H_{2}O$$ (7)

$$O_{2}+2H^{+}+2e^{-}\to H_{2}O_{2}$$ (8)

$$H_{2}O_{2}+2H^{+}+2e^{-}\to 2H_{2}O$$ (9)

$$2H_{2}O_{2}\to 2H_{2}O+O_{2}$$ (10)

Besides the sluggish ORR kinetics, the fabrication cost can also be a problem. Noble metals like platinum have become the most used catalysts in fuel cells due to their high stability and exchange current density. But the high price of platinum (accounting for 55% of the entire fuel cell manufacturing cost) has made it necessary to develop other low-cost, durable, safe, and efficient alternatives to replace platinum catalysts in DMFCs (Litster and McLean, 2004; Cho et al., 2007; Tasic et al., 2009; Zhang et al., 2009; Sharma and Pollet, 2012; Zhang et al., 2017; Gong et al., 2018; Zhang et al., 2019a; Nosheen et al., 2019; Qiao et al., 2019; Martinaiou et al., 2020a; Hou et al., 2020; Li et al., 2021; Wu et al., 2021; Ying, 2021; Shang et al., 2022). Low-platinum catalysts refer to those with a lower Pt content than the commercial Pt/C catalysts and are currently rapidly developing.

The commercial prospects of fuel cells depend on the conversion efficiency of fuels to electricity, so whether the electrode material can continuously generate electrons and drive them towards the cathode has become the key factor. For the design of new cathode catalysts, the electrode materials need to have these features: 1) they must have pores for the reactants; 2) they need to have catalytic effects of breaking the bonds of the fuel and promoting the generation of reactive ions; and 3) they need to have the ability to conduct electrons (Yu and Ye, 2007; Katsounaros et al., 2014; Majlan et al., 2018). The development of cathode catalysts with high catalytic activity for oxygen reduction, strong methanol resistance, and low cost has been an important topic in the research of DMFCs.

Pt is the most used cathode catalyst for ORR, with wide applications in fuel cell vehicles. ORR with Pt/C as catalysts undergoes the four-electron pathway and has the highest energy conversion efficiency. If incomplete ORR happens and takes the two-electron pathway to produce H_2_O_2_, a disproportionation reaction will occur to produce oxygen again, which will seriously affect the energy conversion efficiency of the fuel cell. Electrocatalysts made of Pt are still commonly used because they have the highest activity and stability over time for electrode reactions. However, their commercialization is limited by their high cost and low storage capacity (Hodnik et al., 2012; Kolla and Smirnova, 2015; Zhao et al., 2016b; Wang et al., 2016; Shi et al., 2017; Kwon et al., 2018; Cheng et al., 2019). Many attempts have been made to develop Pt-based catalysts with low Pt loading while maintaining high activity and durability under harsh reaction conditions. The optimization of the nanostructures and the distribution of Pt in the active sites is the key to improving the activity and durability of catalysts. ORR is very sensitive to the surface electrical characteristics of the catalyst as well as its surface atomic arrangements (Tritsaris et al., 2011; Tamaki et al., 2017; Cao et al., 2018; Song et al., 2020). Therefore, studying the surface properties of Pt-based catalysts can effectively modulate the catalytic performance of ORR catalysts.

The reactions in fuel cells consist of two half-reactions, ORR and HOR, and throughout the process of the cathode reactions, the rate-determining step of ORR is the transfer process of the first electron (Joghee et al., 2015; Yu et al., 2019). Under alkaline conditions, many catalysts exhibit good ORR catalytic performance thanks to the first electron transfer process, which requires a low overpotential. Therefore, the main limits to fuel cell energy conversion efficiency are the slow kinetics of ORR as well as high Pt costs. Even if all the Pt reserves (28,000 tons) were depleted and used for fuel cell vehicles, it would only be enough to supply less than 10 million vehicles, not to mention Pt for other uses like jewelry (Brouzgou et al., 2012; Ong et al., 2017). In addition to the high price of Pt and the small amount of Pt available, commercial Pt/C in fuel cell vehicles (FCV) works in an acidic operating environment, where high overpotential will lead to dissolution and deposition of Pt particles, while Pt may also be oxidized, which will lower the stability of Pt/C catalysts. CO poisoning during methanol oxidation can also affect the activity of ORR. In DMFCs, methanol can easily penetrate from the anode to the cathode and induce oxidation reaction on the catalyst surface, resulting in methanol poisoning and the loss of catalyst activity.

**FIGURE 4:**
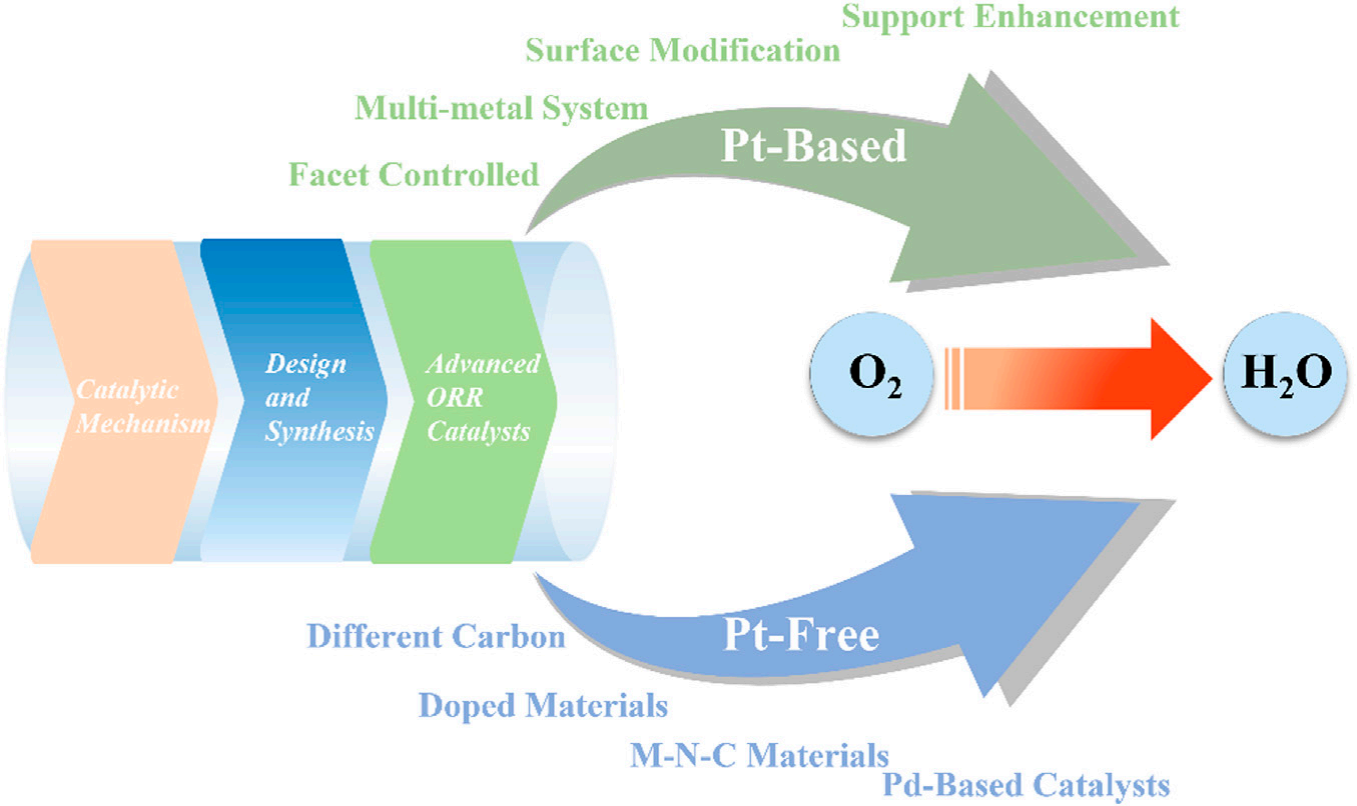
Low Pt-based catalysts and Pt-free catalysts for DMFCs.

The study of novel ORR catalysts used in DMFCs has developed in two ways. The first is to develop Pt-free catalysts to reduce the fabrication cost, and the other is to develop Pt based composite catalysts with lower costs that can maintain the high activity. In recent years, progress has been made in the research of Pt-free ORR catalysts, with excellent ORR activity in alkaline electrolytes comparable to Pt-based catalysts. However, the ORR activity of Pt-based catalysts under acidic conditions is still irreplaceable by Pt-free catalysts at this stage. Therefore, the development of Pt-free-based catalysts with high catalytic activity and stability under acidic conditions is also quite important. Figure 4 shows the strategies to fabricate low Pt-based catalysts and Pt-free catalysts for DMFCs. For Pt-based ORR catalysts, using multi-metal systems like binary and trinary Pt-metal alloying is a good way to reduce the overall cost while maintaining the good activity of Pt-based catalysts. Surface modification and facet control can be utilized to reduce the amount of Pt loading on the surface. 3D nanostructure designs like core-shell structures and nanoframes can effectively increase the activity of the ORR catalysts. For Pt-free catalysts, various materials can be used, such as doped materials, chalcogenides, Pd-based materials, etc. The support can also be optimized to improve the performance of both Pt-based and Pt-free ORR catalysts. N, B, or metal-doped carbon materials are used as supports to increase the overall ORR activity (Nie et al., 2015). A variety of carbon materials such as graphene, graphdiyne, and carbon nanotubes are used to optimize the performance of cathode catalysts for DFMCs. This article provides a comprehensive overview of low- and non-platinum-based catalysts in recent years for the fabrication of low-cost and high-efficiency DMFCs.

## Pt-based catalysts for direct methanol fuel cells cathodes

Pt is not only an expensive and scarce element, but it also has poor stability for ORR. Effective non-precious catalysts require certain properties like electrochemical activity, chemical and mechanical stability, and electrical conductivity. Catalytic reactions are surface reactions where only the atomic energy at the surface plays the catalytic role, while the atoms inside the materials do not participate in the catalytic reaction. Therefore, to obtain the same catalytic activity with a lower amount of Pt, it is necessary to make most of the Pt atoms as surface atoms. The strategies to minimize the loading of Pt in ORR catalysts include Pt-based alloying, Pt-based nanostructure design, and supports-enhanced methods (Alia et al., 2010; Brouzgou et al., 2012; Zheng et al., 2014; Ma et al., 2017; Li et al., 2018; Qu et al., 2018; Zhang et al., 2019b).

Although many electrocatalysts with low Pt content have been tested and characterized for ORR, only a few catalysts have been investigated in DMFCs. As reported by Brouzgou et al. (2012) most of the electrocatalysts have a maximum power density between 0.01 and 0.1 mW μg^−1^, while only some of them can exceed 0.1 mW μg^−1^. Like the anodes in DMFCs, the total Pt loading of the low-Pt cathode for DMFCs remains relatively high. Sakthivel et al. (2010) used surfactants to stabilize Pt nanoparticles and obtained a great maximum performance for Pt electrocatalysts deposited on multi-walled carbon nanotubes (MWCNTs), keeping the total Pt loading at a lower level. The use of surfactants is an important factor in optimizing the even size and homogeneous distribution of nanoparticles. Li et al. (2010) used a Pt-Fe/C (1.2:1) alloy as the cathode with a Pt loading of 1,000 μg_Pt_ cm^−2^ and a maximum power density value of 0.12 mW μg^−1^. This great performance is due to the small particle size and better structure of the Pt-Fe alloy.

In DMFCs, methanol molecules can permeate through the proton exchange membrane to the cathode. Pt metal has poor methanol tolerance because Pt is also highly active for MOR, and intermediates such as CO generated by methanol oxidation can also poison Pt. So, modifying Pt-based catalysts to make them more resistant to methanol is a top research goal. Antolini et al. (2008) studied and summarized the methanol-tolerant Pt-based cathode catalysts for DMFCs. Based on the experimental results, the high activity of the alloys can be attributed to either electronic effects (due to the formation of an alloy that changes the d-band vacancies of the Pt metal) or geometric effects (the formation of an alloy that changes the coordination number and Pt atomic spacing of Pt). While it is, of course, possible for both effects to be present simultaneously. Brouzgou et al. (2012) reported the mass ORR activity of some low-Pt cathodes in the presence or absence of methanol. Among the investigated materials, the carbon-supported Pt-Cr nanoalloy (50 μg_Pt_ cm^−2^) prepared by Yang et al. showed the highest mass activity (Yang et al., 2004). This catalyst exhibited better methanol tolerance due to its disordered surface structure. The Pt_3_Co/C prepared by Colmenares et al. (2007) is also a good ORR electrocatalyst for DMFC, and the catalyst exhibited high mass activity with a value of 0.187 mA and good methanol tolerance. They also investigated Pt_3_Ni alloy catalysts with an activity of 0.135 mA, which had better methanol tolerance than Pt too. Liu et al. investigated a new nanocomposite cathode catalyst consisting of FePc, Pt, carbon black and Nafion (FePc-Pt/C-Nafion), which showed better catalytic activity than commercial Pt/C, both with (0.048 mA) and without methanol (0.086 mA) (Liu et al., 2010b). Based on these results, the excellent tolerance of methanol is the key focus for the development of novel catalyst materials.

### Binary and trinary alloying of Pt-based catalysts

The cell performance of DMFC is usually lower than that of PEMFC using hydrogen as fuel due to the sluggish MOR and ORR reaction kinetics in both the anodes and cathodes, and the Pt loading in the catalyst layer is also significantly higher. In recent years, a variety of Pt-based alloy systems with various compositions have been studied, such as Pt-Pd, Pt-Au, Pt-Ag, etc. (Alcaide et al., 2011; Wang et al., 2011b; Arikan et al., 2013; Cao et al., 2015; Feng et al., 2016; Gowthaman et al., 2018) These binary and ternary alloy catalysts have improved ORR activity and relatively large surface areas. The unique structural properties and composition increase the catalytic activity and further reduce the Pt loading. The design of binary and ternary nanostructures of Pt is a good strategy to develop low-Pt ORR catalysts, which even exhibit better performance than monometallic Pt.

Stamenkovic et al. (2006) investigated the polycrystalline alloys of Pt_3_M (M = Ni, Co, Fe, or Ti) to study the effect of 3d transition metals on the ORR performance of Pt alloys. It was discovered that the activity of this alloy is determined by the surface properties of the 3d metal. They also revealed the correlations between the electrocatalytic on the surface of Pt_3_M, the d-band center, and ORR activity. This correlation exhibits a “volcanic” shape where the highest activity is related to the nature of the 3d metal (Stamenkovic et al., 2007b). To boost the removal of oxygen-containing intermediates, an effective ORR electrocatalyst should have a lower binding energy compared to Pt. They revealed that an optimal ORR catalyst exhibits a weaker binding energy of about 0.2 eV for oxygen-containing species compared to pure Pt (Stamenkovic et al., 2006; Tripković et al., 2010). In this regard, Ni, Co, and Fe are the most effective alloying elements. This trend can help researchers choose the most appropriate alloy components to control the surface chemisorption characteristics of Pt. It was also found that single-crystal Pt_3_Ni (111) exhibited enhanced catalytic activity for ORR, which was 10 times higher than that of commercial Pt (111). The significant increase in catalyst activity is attributed to the presence of Ni, which weakens the adsorption of OH^−^ on the catalyst surface and thus increases the adsorption sites of O_2_ (Stamenkovic et al., 2007a).

**FIGURE 5:**
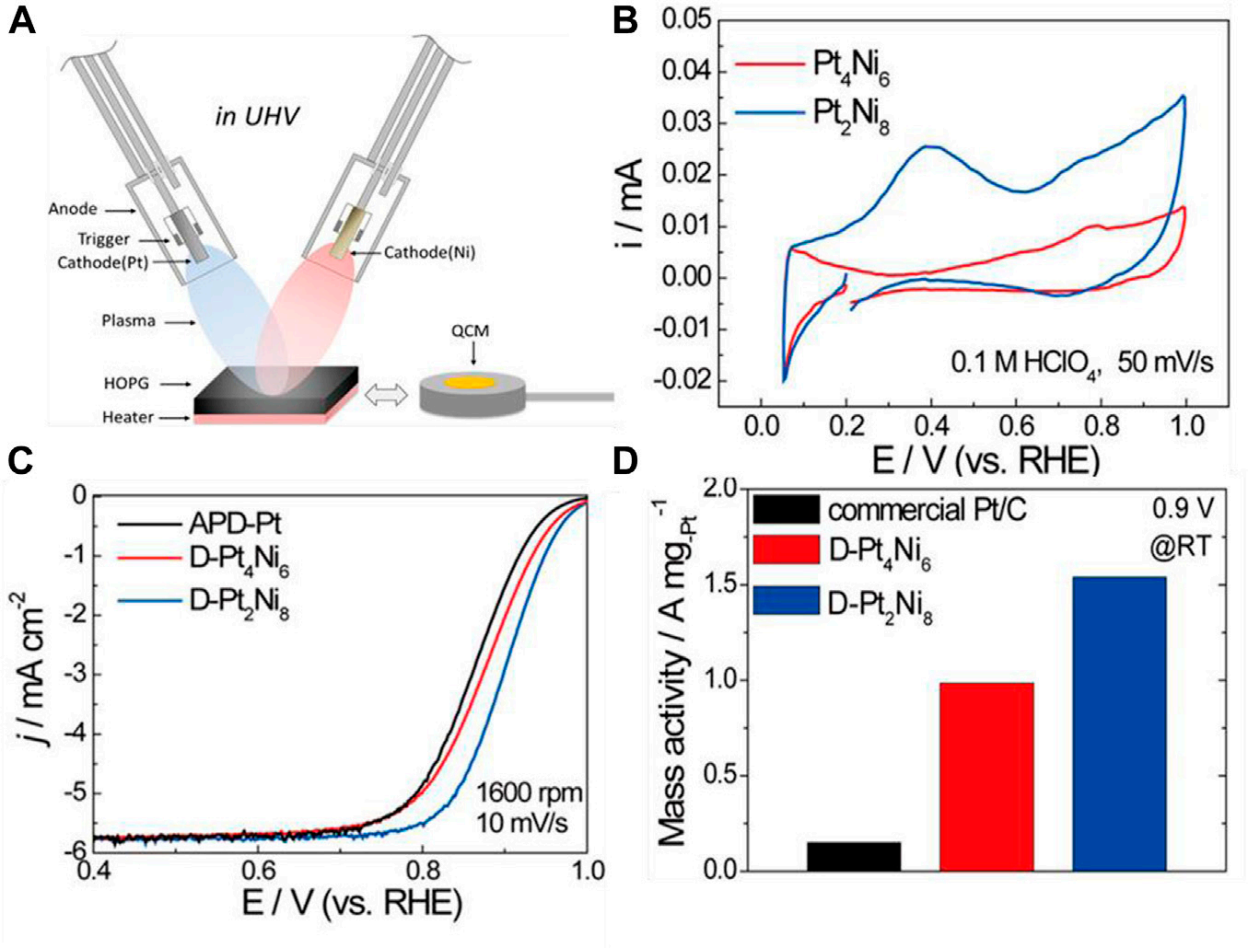
**(A)** Illustration of the arc-plasma deposition of Pt-Ni catalyst; **(B)** cyclic voltammograms (CV) for Pt_4_Ni_6_ and Pt_2_Ni_8_; **(C)** linear-sweep voltammograms (LSV) for the ORR of as-prepared films; and **(D)** Pt-mass activities for the as-prepared films and commercial Pt/C. Reproduced with permission (Todoroki et al., 2015). Copyright 2015, American Chemical Society.

As for effective Pt-Ni alloy catalysts, Antolini et al. (2005) prepared carbon-supported Pt-Ni alloys with sodium borohydride at room temperature and tested their performance for DMFCs for the first time. The obtained catalysts worked as both cathodes and anodes, and the performance of Pt-Ni as a cathode is higher than that of Pt-Ni as an anode. The catalysts were prepared with a Pt to Ni ration of 90:10 or 70:30. As a cathode material, Pt_90_Ni_10_ is superior to Pt in both mass activity and specific activity. The enhanced performance of Pt-Ni as cathode is attributed to the electronic effect of ORR and better methanol resistance related to the alloy. Todoroki et al. (2015) investigated Ni-Pt alloy nanoparticle-stacking thin films (NPSTF) as a novel ORR electrocatalyst. The Ni-Pt was deposited *via* the arc plasma deposition method. Arc plasma is created by a gas arc discharge and can generate nanosized particles with the high temperature discharge (Gerdeman and Hecht, 2012). The mass activity of the as-synthesized Pt−Ni NPSTF is 10 times higher than that of commercial Pt/C catalysts. Pt−Ni NPSTF exhibits a significant ORR activity enhancement, which is attributed to the underlying Ni atoms’ modification of the surface Pt-rich layer and the enhanced active surface area caused by the stacking of PtNi nanoparticles, as shown in Figure 5.

Zou et al. (2015) synthesized an ordered PtNi (O-PtNi/C) intermetallic compound supported by carbon from the conversion of disordered PtNi nanoparticles. The nanoparticles were converted by a heat treatment method at 550–600°C under a H_2_/N_2_ atmosphere. The intermetallic nanoparticles were characterized and showed increased mass and specific activity. At 0.85 V, the specific activity of the PtNi nanoparticles is about 6 times that of commercial Pt/C and 3 times that of disordered PtNi alloy. The stability of the as-prepared catalyst may be related to the structural changes during the heat treatment.

### Pt-based core-shell structures

Besides Pt alloying, designing structures at the nanoscale is also an effective way to disperse Pt on the surface to the greatest extent and to reduce the overall Pt loading. Pt alloying can be combined with appropriate core-shell structures or nanoframes to maximize the performance of the catalysts (Wu et al., 2019). Pt-based core-shell nanostructures consist of a core made with metals, metal oxides or metal nitrides and a thin layer of Pt grown on the surface (Zheng et al., 2018). They have improved Pt utilization, catalytic activity, and stability. Yang et al. (2016) used density functional theory (DFT) calculations to screen binary and ternary Pt-shell electrocatalysts made with non-precious elements for ORR. By calculating the adsorption intensity of O on nanoalloy clusters using DFT, the trend of ORR activity of Pt-shell electrocatalysts may be determined. The equilibrium geometry of a three-layer Pt_42_M_12_N_1_ (M, N = Fe, Co, Ni, and Cu) cluster model has an icosahedral (Ico) structure. To maximize ORR activity, all atoms in the surface layer are Pt atoms, and atoms in the subsurface layers are transition metals to lower the amount of Pt utilized. If M is the same as N, the systems are binary Pt_42_Fe_13_, Pt_42_Co_13_, Pt_42_Ni_13_ or Pt_42_Cu_13_ clusters. When M is not the same as N, the systems are ternary Pt-shell clusters, such as Pt_42_Cu_12_Ni_1_ and Pt_42_Fe_1_Co_1_. The surface of the Ico structure includes 20 facets, so the adsorption on these highly symmetric nanoclusters is worth studying. For each triangular face, six different adsorption sites were found. In binary Pt-M (M = Fe, Co, Ni, and Cu) electrocatalysts, Pt_42_Fe_13_ has the strongest ORR activity. While Pt_42_Fe_12_Cu_1_ was the most effective catalyst in ternary Pt-M-N systems. Yang et al., 2021b reported a bimetallic Pt-metal core-shell structure with good ORR activity. The Pt_3_Co@Pt core-shell structures had a mass activity of 0.71 mA mg_Pt_^−1^ and specific activity of 2.75 mA mg_Pt_^−1^ attributed to the well-defined core-shell structure.

Kuttiyiel et al. (2012) reported an electrocatalyst with low-Pt monolayer and a bimetallic IrNi core on carbon support (Pt_ML_/IrNi/C) using the potential shift of a Cu layer synthesized at a negative potential. The Pt mass activity of the as-prepared electrocatalysts in the scaled-up synthesis is three times higher than that of the commercially available Pt/C electrocatalysts. The electronic and geometric effects of the IrNi substrate led to enhance activities. The experimental results are supported by DFT using a spherical model, demonstrating an effective approach to improve the activity of cathodic oxygen reduction.

**FIGURE 6:**
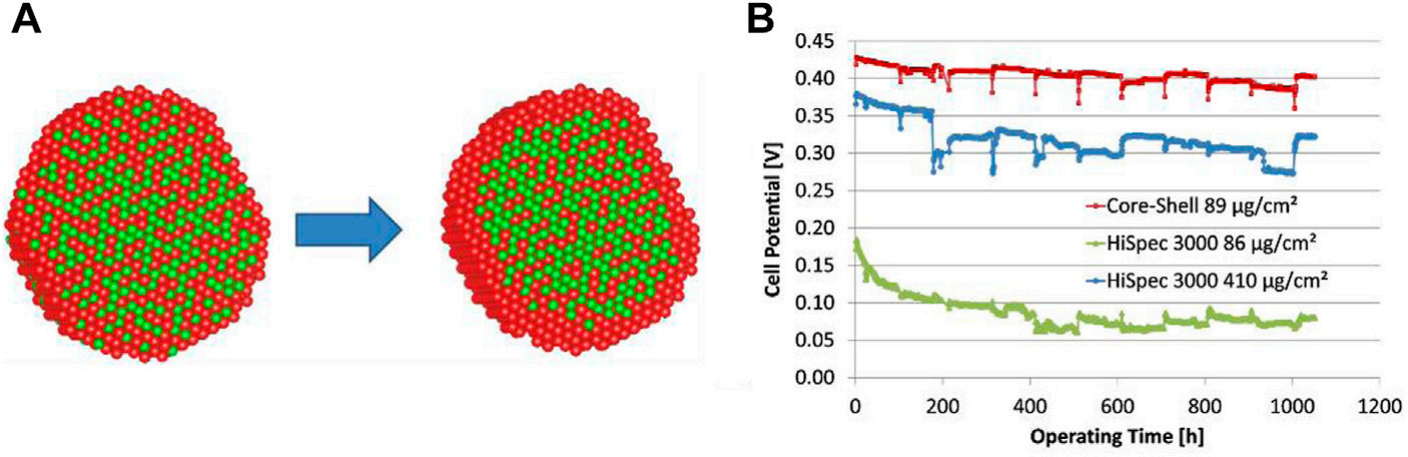
**(A)** Model of the core−shell structure before (left) and after (right) electrochemical tests; **(B)** Cell potential during long-term operation at 70°C in 0.75 M methanol solution. Reproduced with permission (Glüsen et al., 2019). Copyright 2019, American Chemical Society.

Glüsen et al. (2019) fabricated a PtNi core-shell structure for reduced Pt content in DMFCs. The core-shell catalyst initially exhibited similar performance to commercial Pt. But after operation, a Pt-rich shell forms with a 0.7 nm thickness, which corresponds to 3 Pt atom layers. This Pt shell led to increased DMFC performance, which was comparable to a cathode consisting of five times the Pt amount. Figure 6A shows the difference in the Pt shell before and after the electrochemical tests, which could be attributed to leaching of the Ni atoms from the core. Figure 6B shows the stability test over a long time, and the performance showed no decay even after 1,000 h of operation due to the robust durability of the core−shell catalyst. In the future, it would be beneficial to expedite such a structure alteration so that the catalyst can achieve the optimized performance in a shorter time.

Nanoframes with 3D surfaces can achieve higher catalytic activity with lower Pt content because the atoms on the inner and outer surfaces can contribute to catalysis at the same time, resulting in a much higher utilization of active atoms. Structural control at the atomic level can precisely and efficiently tune the catalytic performance of the material, thus improving activity and durability. Chen et al. (2014) used a unique air etching method to synthesize polymetallic Pt_3_Ni nanoframes as an ORR catalyst, and they conducted an in-depth study on the structure and formation process of the materials. Characterization results by elemental mapping, X-ray photoelectron spectroscopy (XPS) and transmission electron microscopy (TEM) demonstrated that a 3D nanoframe structure was formed with Pt-atom-rich surfaces due to the limited etching effect of O_2_ on Ni. The obtained Pt_3_Ni catalyst has a very high catalytic activity for the ORR, with a mass activity 22 times higher than that of commercial Pt/C. The method is practical for the synthesis of polymetallic nanoframes and can be used to prepare frame structures such as PtRuNi, PtPdNi, etc. The fabrication of Pt-based nanoframes has good prospects for scale-up applications.

### Combine with transition metal nitrides, oxides and carbides

Transition-metal nitrides (TMN), oxides, and carbides have ORR activities and can be used in Pt-free catalysts, as will be discussed below, but they can also be combined with Pt-based materials to improve the overall activity and durability of the composite catalyst (Yuan et al., 2022). Carbide-based Pt alloy composites are fabricated in various forms, such as core–shell structure, nanoparticles, and single atoms. With the excellent durability and large specific surface area of carbides, these compounds have great potential in ORR catalysis (Li et al., 2019a). They not only reduce noble metal usage but have also been shown to enhance the intrinsic activity and durability of Pt in ORR (Park et al., 2014; Zhang et al., 2014; Zhao et al., 2015; Lin et al., 2019). Transition metal oxide-supported electrocatalysts are regarded as one of the most promising electrode catalyst alternatives due to their great stability than carbon under various operating conditions. The introduction of metal oxides can provide anchor points for the nucleation and growth of Pt but also increases the exposure of active interfaces for efficient catalytic reactions (Liu et al., 2017; Song et al., 2018; Chen et al., 2021). In addition, the strong interfacial active band of the metal oxide itself and the interaction between Pt and metal oxide can not only turn Pt into an electron-rich state but also increase the surface energy and stabilize the growth size of the material. The goal of speeding up the chemical reaction is met while the amount of Pt in the electrocatalyst is decreased (Bertram et al., 2020; Xu et al., 2020; Yang et al., 2021a; Chen et al., 2021; Valizadeh et al., 2022).

**FIGURE 7:**
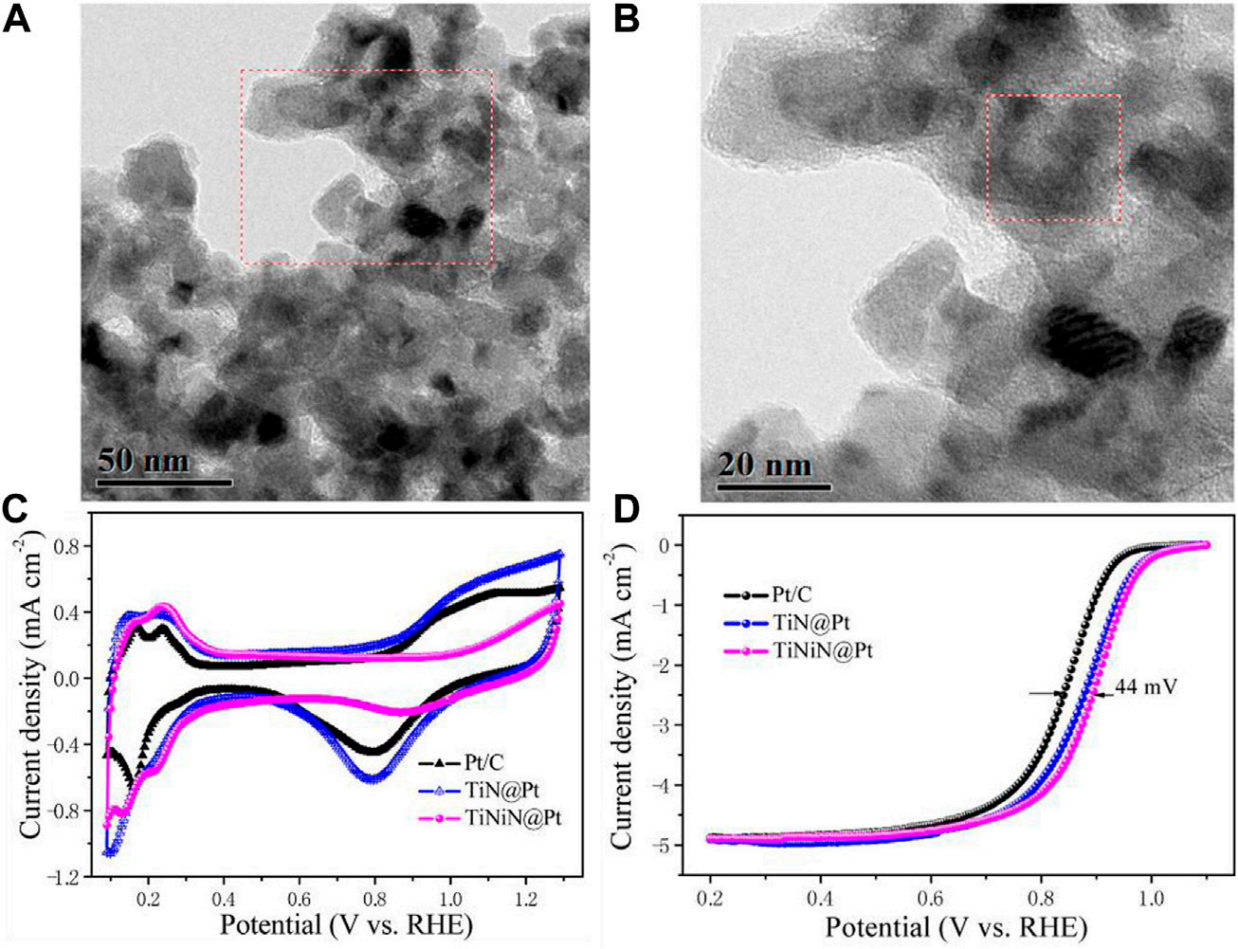
**(A,B)** TEM images of TiNiN@Pt catalyst; **(C,D)** CV curves and ORR polarization curves of the as-synthesized samples. Reproduced with permission (Tian et al., 2016). Copyright 2016, American Chemical Society.

In addition, transition metal nitrides with high electrical conductivity and thermal stability are suitable ORR electrocatalysts (Ham and Lee, 2009; Cao et al., 2013; Kumar et al., 2013; Youn et al., 2013). The formation of nitrides alters the electronic structure of the catalyst, but the interactions within TMN can also enhance the adhesion of the supported noble metals, thus enhancing the electron transfer of the catalytic process (Zhong et al., 2006; Xia et al., 2008; Cui et al., 2013; Yang et al., 2013; Cui et al., 2014). Tian et al. (2016) synthesized an effective TMN catalyst with TiNiN as the core coated with a layer of Pt . The TiNiN@Pt catalyst showed great ORR catalytic activity, with a more than 4-times increase in mass activity compared to commercial Pt/C. It also possessed long-term durability within 10,000 potential cycles due to the strong binding between Pt nanoparticles and TiNiN. Figures 7A,B shows the TEM images of the particles, and Figures 7C,D shows the CV curves and ORR LSV curve of the as-synthesized catalyst. This catalyst avoided the corrosion problem of conventional carbon supports by using highly stable transition metal nitride and successfully enhanced the durability of the Pt/TMN composites.

## Platinum-free catalysts for direct methanol fuel cells cathodes

The parasitic reactions on the cathode caused by the sluggish ORR kinetics and methanol crossing should be minimized to further develop advanced cathodic catalysts for DMFCs. Catalysts such as Ag (Park et al., 2016), Au (Li et al., 2019b), Pt (Zhu et al., 2021), Pd (Ejaz and Jeon, 2018), and nitrogen-doped carbon nanotubes (NCNTs) (Ejaz and Jeon, 2018), have all shown enhanced ORR activities. Based on current progress in this field, it is necessary to explore platinum-free cathodic catalysts with high performance and methanol resistance. In recent years, breakthrough progress has been made in the research of Pt-free cathodic catalysts. New materials including transition metal chalcogenides, transition metal nitrogen-carbon composites, and transition metal macrocyclic compounds all show great catalytic activity for ORR.

### Transition metal chalcogenides

As a new type of Pt-free oxygen reduction catalyst, transition metal sulfide compounds have attracted research attention because of their abundant sources and good stability in acidic media. The most studied transition metal sulfide compounds are mainly Ru-based materials serving as alternative Pt-free catalysts. The transition metal sulfide compounds can be divided into two types: binary compounds and ternary compounds. Brouzgou et al. (2012) summarized the methanol resistance of the transition metal chalcogenides. The activity of the Co-Se carbon-loaded ORR catalyst remained constant in the presence of methanol (Nekooi et al., 2010). Pd-Se catalysts also showed great activity and good ORR tolerance. According to Jeng et al. (2011), the performance of DMFC using RuSe/CNT as the cathode catalyst was better than that of Pt/C, depending on the type of carbon support.

Ru-based and Ir-based chalcogenides are also promising ORR catalysts with high methanol tolerance in acidic media. Lee et al. reported the iridium-selenium (Ir-Se) system with high ORR catalytic activity, which may be due to the bimetallic interaction effects (Lee et al., 2007b). Alonso-Vante et al. (2002) used *in situ* extended X-ray absorption fine structure (EXAFS) to study Ru_x_X_y_ (X = S, Se, and Te) compounds. It was found that the catalyst had a Ru atomic nucleus with triangular coordination and direct metal-metal bonding. The catalytic activity depends on Ru atomic cluster size and O_2_ interaction. Among the as-synthesized chalcogenides, Ru_x_Se_y_ binary compounds are the most catalytic. To boost ORR catalytic activity and methanol tolerance, transition metal sulfide compounds need improvements in compositional and structural properties. Recently, composite materials with cobalt and sulfur supported by carbon have also made progress. Zhang et al. synthesized CoS_0.197_-C composite as efficient ORR catalyst from Co nitrilotriacetic acid (Co-NTA) polymer precursor (Zhang et al., 2019c). The unique 1D porous structure provides more active sites and contributes to the ORR catalytic performance; it also enhances the electron transport between the catalyst and the carbon support.

**FIGURE 8:**
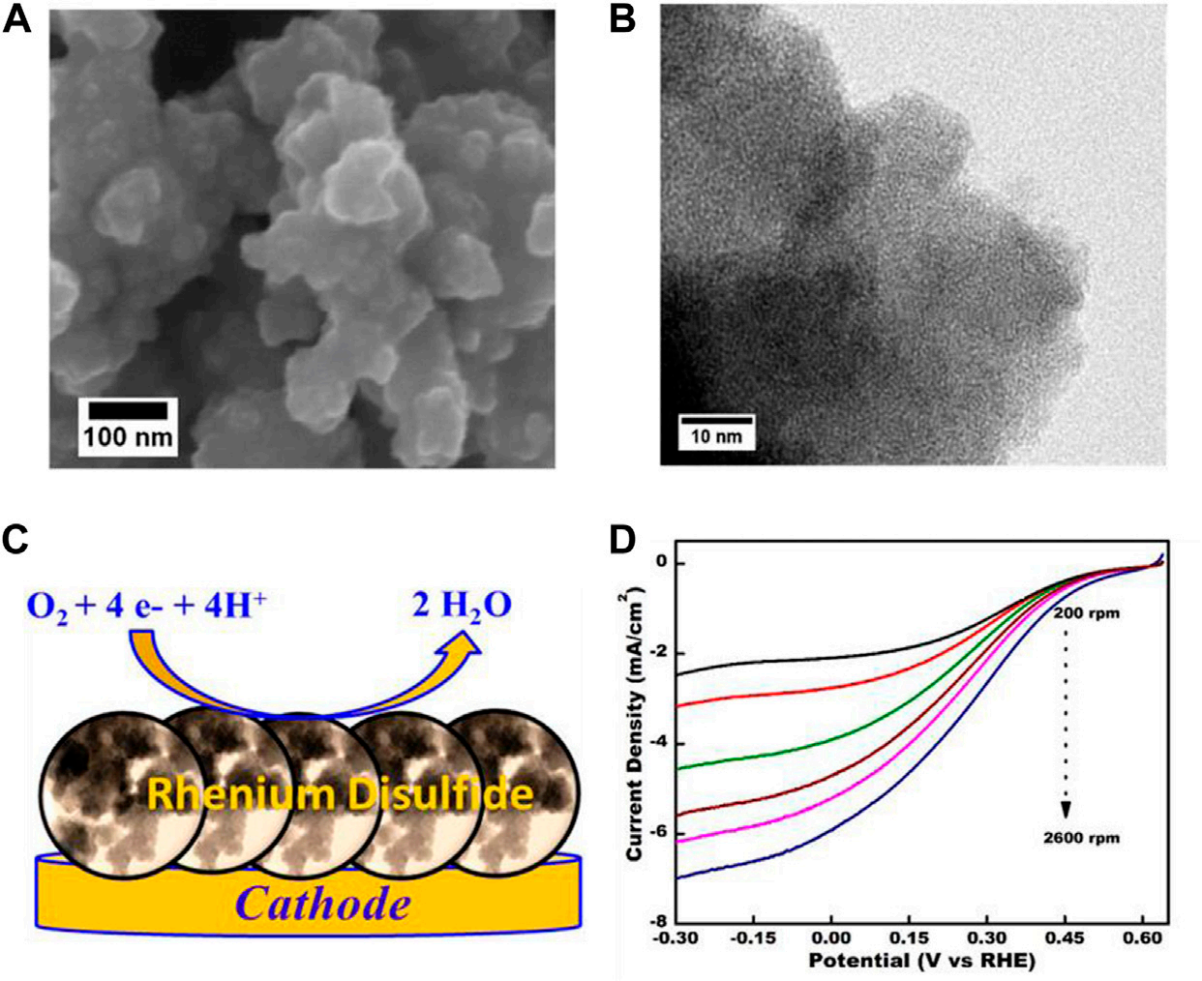
**(A,B)** FESEM and TEM images of the as-synthesized ReS_2_ catalyst; **(C)** The illustrate of ORR process on the ReS_2_ cathode; **(D)** The ORR curves of the as-synthesized catalyst at different rotation rates. Reproduced with permission (Radhakrishnan et al., 2019). Copyright 2019, American Chemical Society.

Radhakrishnan et al. (2019) synthesized amorphous ReS_2_ nanosheets as methanol-tolerant ORR catalysts. Figures 8A,B show the FESEM and TEM images of ReS_2_, respectively. Figure 8C shows the illustration of the ORR process on the surface of the catalyst. As shown in Figure 8D, the ReS_2_ electrode obtained increased current density within the potential range of 0.6 to −0.3 V at higher rotation rates. More importantly, the ReS_2_ electrode exhibited 3 times higher current density than MoS_2_ under the same rotation rates. The catalyst also outperformed commercial Pt/C in terms of long-term durability and methanol tolerance.

### Ir- and Ru-based catalysts

Studies on Ru-based and Ir-based compounds have made some progress, but as noble metals, Ru- and Ir-based catalysts are still costly. Like Pt-based catalysts, alloying would be a good solution to reduce the fabrication cost. Both Ir and Ru work well with Co as composites for enhanced ORR activity. Ir_x_Co_1−x_ alloys exhibited good ORR activity and better methanol tolerance compared with Pt/C catalysts (Lee et al., 2007a).Liu et al. (2021) introduced Ru into Co clusters with ZnRuCo ZIF as a precursor, and synthesized Co encapsulated with nitrogen-doped carbon (α-Ru@Co/CN). Although in this work the obtained catalyst was tested in direct ethanol fuel cells, the ORR activity of the clusters was remarkable with a half wave potential of 0.908 V vs. RHE. The performance was attributed to the strain and ligand effects of the encapsulated structure and the introduction of the Ru element.

**FIGURE 9:**
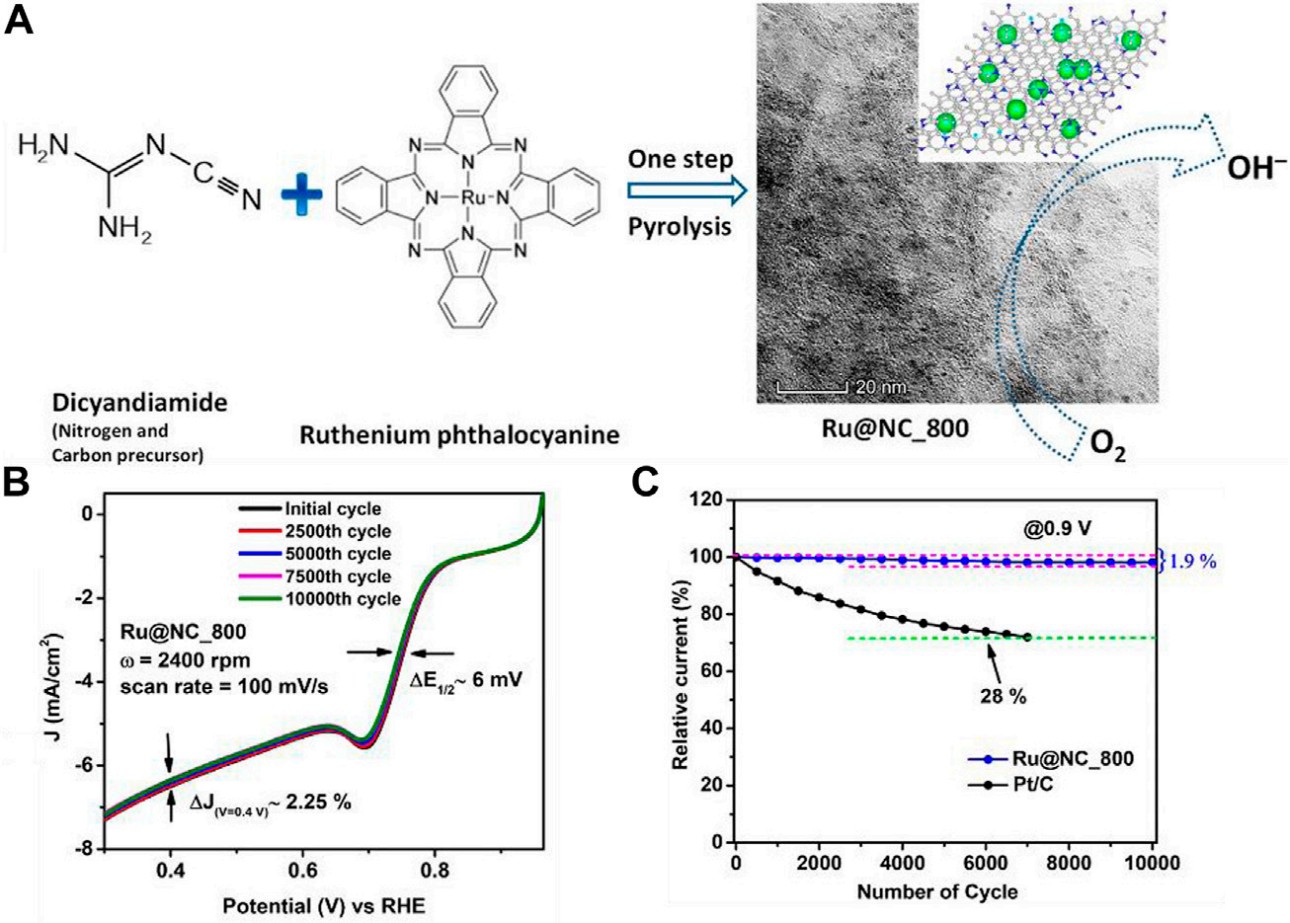
**(A)** Illustration of the synthesis route; **(B)** activity of the as-synthesized catalyst for different cycles of tests; and **(C)** the stability comparison of Ru@NC_800 and commercial Pt/C. Reproduced with permission (Bisen and Nanda, 2021). Copyright 2021, American Chemical Society.

Bisen and Nanda (2021) synthesized a Ru nanoparticle-loaded carbon structure for ORR. The illustration for the synthesis is shown in Figure 9A. The Ru@NC catalyst was synthesized by one-step pyrolysis of the dicyandiamide and ruthenium phthalocyanine at 800°C. The Ru@NC exhibited great ORR activity (Figure 9B), which was due to the improved 4-electron transfer *via* the inner-sphere electron transfer (ISET) mechanism. Figure 9C shows the stability of the as-synthesized catalyst compared to Pt/C catalyst. This work illustrates the effects of synthesis temperature and rotation speeds on Ru@NC catalyst performance. Moreover, the Ru@NC largely suppresses intermediate HO_2_^−^, which leads to high stability over 10,000 cycles and surpasses commercial Pt/C.

### Metal-nitrogen-carbon catalysts

Transition metal-nitrogen-carbon (M-N-C) complexes are good non-precious metal ORR catalysts due to their high activity and resistance to methanol crossover effects. As a possible substitute for precious metal-based cathode catalysts, M-N-C materials have caught the attention of researchers and are expected to be a key component of the most advanced cathode catalysts. The study of M-N-C compounds for ORR began in 1964, when Jasinski (1964) revealed that cobalt phthalocyanine possessed ORR activity. Yeager (1984) discovered the pyrolysis of M-N_4_-macrocycle precursors to produce the first M-N-C composite for oxygen reduction. Many effective ORR catalysts based on M-N-C have been created since then.

**FIGURE 10:**
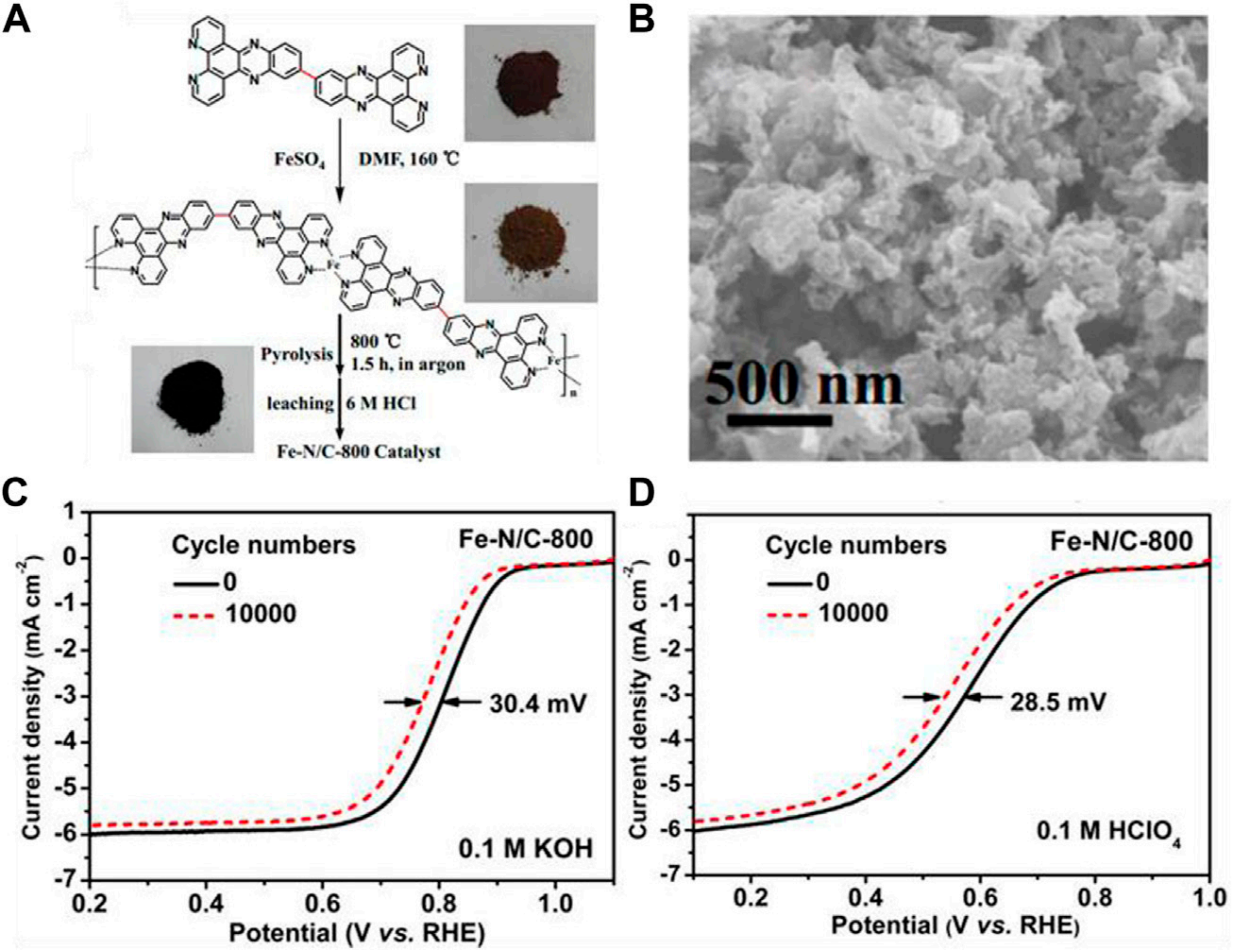
**(A)** Illustration of the synthetic process of the FeN/C-800 catalyst; **(B)** SEM image of the as-prepared catalyst; **(C,D)** Stability test of the as-prepared catalyst in O_2_-saturated 0.1 M KOH and 0.1 M HClO_4_. Reproduced with permission (Lin et al., 2014). Copyright 2014, American Chemical Society.

Lin et al. (2014) utilized a conjugated organic molecule containing pyridine nitrogen as a ligand to synthesize a metal coordination polymer. They ligated the polymers with Fe^2+^ ions and then pyrolyzed them to generate a self-supported catalyst (Fe-N/C) with a high nitrogen and iron doping density. The synthesis route and SEM image of this catalyst is depicted in Figures 10A,B. The catalysts calcined at 800°C possessed a relatively high density of surface-active sites with outstanding ORR activity. Although their specific surface area was not particularly high, their catalytic activity under acidic and basic conditions was comparable to that of Pt/C. What’s more, the Fe-N-C structure was more resistant to methanol than Pt/C. Figures 10C,D show the activity of the Fe-N/C-800 before and after stability tests, in alkaline and acidic media, respectively. The results indicate that the composite materials have great durability in solutions with a wide range of pH.

Recently, Bisen et al. (2021) reported a simple method to conduct pyrolysis of cobalt phthalocyanine (CoPc) and successfully synthesized Co-N-C catalysts without any chemical waste. The Co-N-C catalyst demonstrates improved ORR activity, it was also effective in alkaline media. Martinaiou studied the activity of Fe-N-C under the effect of methanol and tested their degradation process. The results showed that adding methanol has no negative effect on the durability of the catalyst, indicating that Fe-N-C is a stable ORR catalyst with good methanol tolerance (Martinaiou et al., 2020b).

### Pd-based catalysts

Pd-based catalysts are great substitutes for Pt-based ORR catalysts (Sanij et al., 2021). To address the issue that Pt-catalysts are easily poisoned in DMFC, Zhang et al. (2020) fabricated Pd-Te hexagonal nanoplates (HPs) as an effective ORR catalyst. The catalyst consists of an ordered arrangement of Pd atoms in the Pd-Te nanoplates that supply active sites for the reactions. As a result, Pd-Te HPs/C exhibited promising ORR activity and excellent stability over more than 50,000 s of chronopotentiometry, with little activity degradation and restricted structure/composition alterations. In DMFC devices, Pd-Te HPs/C outperforms commercial Pt/C in terms of methanol tolerance and antipoisoning stability. DFT simulations confirmed that the unique surface arrangements of the obtained catalysts have an intrinsic contribution to the great performance of the catalyst. Pd-Te HPs’ high activity and superior methanol tolerance ensure their viability as possible electrocatalysts for DMFC and beyond. This work provides a novel method for constructing and developing 2D Pd-based catalysts for enhanced activity towards ORR.

### Porphyrin-based macrocyclic compounds

Porphyrin-based transition metal macrocyclic compounds are considered as potential electrocatalysts for ORRs in DMFCs. Based on literature, the performance of these catalysts is unstable in acidic media, but it can be significantly improved by pyrolysis (Gojković et al., 1999). A cobalt tetramethoxyphenylporphyrin (CoTMPP) precursor was pyrolyzed to fabricate an ORR catalyst with high methanol tolerance (Wang et al., 2011a). The current density of the CoTMPP/C-pyrolyzed electrocatalyst in the presence of ethanol was consistent with that in the absence of methanol, indicating the great methanol tolerance of the ORR catalyst. The activity of the as-synthesized catalyst is related to the temperature of the pyrolysis and the rotation rates. A higher rotation rate at 700°C contributed to better oxygen reduction activity.

Piela et al. (2010) examined six different non-precious catalysts as cathodes for fuel cell applications, including pyrolyzed CoTMPP. They used non-precious metals as cathodes and Pt-Ru/C as anodes to test the performance of the fuel cells by rotating disk electrode (RDE). CoTMPP exhibited high ORR selectivity and performance, which presented a N_4_-metal structure at 500–700°C. CoTMPP shows great potential value for mixed-reactant feed DMFCs that require high ORR selectivity. The performance of pyrolyzed porphyrin-based ORR catalysts greatly depends on the conditions of the heat treatment. It is generally believed that heat treatment can enhance both the activity and stability of these electrocatalysts. For DFMCs with porphyrin-based macrocyclic compounds as cathodes, however, the total fabrication cost is not low because the raw materials are expensive.

### Transition-metal carbides, nitrides and oxides

Combining transition metal with C and N to form metal carbides (TMC) and nitrides can change the properties of metal centers, thus improving their catalytic performance. The Pt-like behavior of tungsten carbide was observed earlier by Levy and Boudart in several catalytic reactions, which motivated the early studies on the properties of metal carbides and nitrides (Levy and Boudart, 1973; Chen, 1996). TMC or TMN are formed by doping C or N atoms into the interstitial transition metal network, respectively. Abdelkareem et al. (2021) summarized the progress of TMC and TMN and their performances in fuel cells. Since there are various preparation methods for TMC and TMN, which have a high similarity in the structures. For example, TiC nanocrystals synthesized by a simple sonication method can efficiently improve the activity of ORR compared with native TiC, as reported by Wang et al. (2020) With abundant defects, oxygen vacancies, and a large pore volume, the as-prepared catalyst also exhibited good stability and methanol tolerance. This increased activity of atomically thin TiC was related to the high specific surface area and abundant oxygen vacancies, which together promote oxygen adsorption and ORR rate.

TMN is usually more stable than TMC under extreme working conditions; therefore, it is more widely used as an ORR catalyst or catalyst carrier. Yuan et al. (2020) reported the preparation of nanoparticulate zirconium nitride (ZrN) using the urea–glass method that works great in alkaline media. The ORR performance of ZrN tested after cycling in 0.1 M KOH at 1,000 rpm had better stability than Pt/C catalysts. The high activity was attributed to the moderate temperature in the urea–glass method, which avoided the nanoparticle aggregation that occurs during the preparation of ZrN from ZrO nanoparticles treated with NH_3_ at 1,200°C. In addition, the authors demonstrate that the thin layer of ZrO generated on the surface of ZrN nanoparticles due to their exposure to air acts as a protective layer and would not hinder electron transfer.

## Carbon supports for cathode catalysts in direct methanol fuel cells

Although many type of non-precious metals have been developed as alternative catalysts for ORR, increasing their stability and methanol tolerance are still a great challenge (Yu et al., 2010a). Therefore, the search for a support that can enhance the durability of the catalyst for ORR has attracted a lot of attention. ORR catalysts are usually composed of various carbon-based supports such as graphite, graphene, carbon nanotubes, ordered mesoporous carbon, etc.

### Doping carbon by heteroatoms

The doping of other elements in carbon materials can change the structure of them, thus affecting their hydrophilicity, electrical conductivity, catalytic activity, etc. Progress has been made in recent years on the addition of heteroatoms, such as B (Yang et al., 2011), N [Gong et al., 2009; Liu et al. (2010a), Yu et al., 2010b], S Yang et al. (2012b), Se Yang et al. (2012b), P (Liu et al., 2011); Yang et al. (2012a), and F (Sun et al., 2013) to modulate the intrinsic properties of carbon materials. Besides the common N-doped materials, other doped carbon catalysts such as B, P, and S also have great ORR catalytic performances. The binary or ternary doped carbon materials such as B-N, N-P, and N-S all have good ORR catalytic activity (Wang et al., 2012; Zheng et al., 2013). Although these catalysts exhibit good catalytic activity under alkaline conditions, their activity under acidic conditions is not as good.

Among the various dopants mentioned above, nitrogen-doped carbon catalysts are the most studied. The N-bonded graphite structure has N atoms instead of carbon atoms. As a result, they have the same structure as graphite carbon atoms, but they introduce more electrons into the off-domain Π-system. Pyridine N can also exist in an oxidized form. These different N functional groups usually coexist, and their concentrations can actually be adjusted (Jin et al., 2011). Among these N-functional groups, pyridine, pyrrole, and graphitic N are usually considered to contribute to the ORR catalytic activity. Some researchers have suggested the involvement of pyridine N in ORR activity (Lee et al., 2010), while others have proposed graphitic N (Luo et al., 2011). One study suggested that graphite and pyridine N sites are interconvertible in ORR (Kim et al., 2011). In addition to the type of nitrogen sites, the number of carbon edge sites and total N content were also found to affect the ORR activities of the materials (Choi et al., 2012).

In general, N/C is prepared mainly by three different pathways. The first method is the *in-situ* introduction of nitrogen atoms into the carbon skeleton through a process such as chemical vapor deposition (CVD) to form graphite planes (Wei et al., 2009; Reddy et al., 2010). This method is usually not suitable for large-scale applications. The second method is post-heating graphitic carbon, including nitrogen-containing materials such as carbon nanotubes, graphene, and fullerenes (Wang et al., 2009a; Gao et al., 2013). The last method is the direct pyrolysis of nitrogen-containing structures, such as graphitic carbon nitride (Zheng et al., 2011), melamine foam (Lee et al., 2013) and polymer backbones (Zhao et al., 2012). This last method is currently the most popular one due to its simple and manageable preparation technique. However, direct pyrolysis at high temperatures often leads to significant loss of active N species and an inability to control the internal pore structure, resulting in limited ORR active site formation and poor migration properties (Nie et al., 2015).

### Carbon nanotubes

Carbon nanotubes (CNTs) can help increase fuel cell performance. For example, Pt can be attached to the inner and outer walls of CNTs, which could lead to an improvement in the ORR catalytic performance of Pt/CNT composites (Zhao et al., 2016a; Wang et al., 2023). But sometimes the nitrogen-doped CNTs can serve as ORR catalysts themselves. Gong et al. (2009) recently found that vertically aligned nitrogen-doped carbon nanotubes (VA-NCNTs) can serve as very effective metal-free ORR electrocatalysts. Well-arranged carbon nanotubes with good electrical and mechanical properties, as well as excellent thermal stability, make them suitable electrode materials under harsh environmental conditions. Based on DFT calculations, the improved catalytic activity was attributed to the electron accepting ability of nitrogen atoms.

### Graphene

The theoretical specific surface area of graphene is 2,630 m^2^ g^−1^, which is much higher than that of carbon black and carbon nanotubes (Hanaei et al., 2016). Due to its huge surface area and improved catalyst dispersion, graphene becomes a promising metal support for ORR catalysts. Chen et al. (2019) developed a single-atom electrocatalyst supported on nanographene for enhanced ORR activity. The catalyst had single Fe atoms anchored by N-doped carbon loading on reduced graphene oxide (Fe-N-NG/RGO). Compared to traditional carbon supports in ORR materials that is generally highly graphitized, the Fe on nanographene had bigger interlayer spacing for better diffusion of O_2_ molecules. The as-prepared catalyst can achieve great ORR activity by the transport of O_2_ to active sites of Fe-N species. Dong et al. (2017) also utilized N-doped reduced graphene oxide to support binary transition metal nitrides for enhanced ORR activity. They found that N-RGO supported transition metal nitride was much more active than the two components alone, indicating that there was a strong synergistic effect to promote the overall activity between the two materials.

## 5 Perspectives

Over the past decades, nanoscience and nanotechnology have provided many opportunities for the rapid development of novel electrode nanomaterials. In addition, the synthetic strategies of new materials provide functionality for wider applications of these materials. In this article, Pt-based catalysts and Pt-free-based catalysts for DMFCs are discussed based on the highlights of different synthesizing methods. In the past, research on ORR catalysts mainly focused on reducing the Pt content and improving the catalytic performance. As the research on DMFC becomes more and more practical, the preparation of cathode catalysts with high methanol resistance also becomes necessary. Meanwhile, with the increase in research interest on Pt-free-based catalysts, their substitution for Pt-based catalysts becomes possible.

Methods such as Pt alloying, core-shell nanostructuring, and combining with TMNs greatly reduce the Pt loading while improving the overall ORR performance of Pt-based catalysts. Pt-free catalysts, such as Pd-based materials, porphyrin-based macrocyclic compounds, transition metal chalcogenides, and M-N-C catalysts have good ORR performance under alkaline conditions, but their performance under acidic conditions is still not ideal. The applications of Pt-free catalysts in practical conditions are still worth to be explored in the future. Both types of catalysts should be combined with carbon supports to facilitate the transport of charged particles. More advanced development on DMFC electrode catalysts will be based on in-depth understanding of the intrinsic structure and distribution of active centers of catalysts. While controlling the cost of Pt in the fabrication process of the cathode materials, the utilization rate of active centers also need to be improved. These novel design strategies help improve the activity and stability of the DMFC cathode and lay a solid foundation for the large-scale application of new energy fuel cells.
